# Quantification of Cognitive Function in Alzheimer’s Disease Based on Deep Learning

**DOI:** 10.3389/fnins.2021.651920

**Published:** 2021-03-17

**Authors:** Yanxian He, Jun Wu, Li Zhou, Yi Chen, Fang Li, Hongjin Qian

**Affiliations:** ^1^One Departments of Cadre Ward, General Hospital of Southern Theater Command, PLA, Guangzhou, China; ^2^Branch of National Clinical Research Center for Geriatric Diseases, Chinese PLA General Hospital, Guangzhou, China; ^3^Three Departments of Cadre Ward, General Hospital of Southern Theater Command, PLA, Guangzhou, China; ^4^Two Departments of Cadre Ward, General Hospital of Southern Theater Command, PLA, Guangzhou, China; ^5^Four Departments of Cadre Ward, General Hospital of Southern Theater Command, PLA, Guangzhou, China

**Keywords:** Alzheimer’s disease, quantification of cognitive function, deep separable convolution, channel pruning, convolutional neural network

## Abstract

Alzheimer disease (AD) is mainly manifested as insidious onset, chronic progressive cognitive decline and non-cognitive neuropsychiatric symptoms, which seriously affects the quality of life of the elderly and causes a very large burden on society and families. This paper uses graph theory to analyze the constructed brain network, and extracts the node degree, node efficiency, and node betweenness centrality parameters of the two modal brain networks. The T test method is used to analyze the difference of graph theory parameters between normal people and AD patients, and brain regions with significant differences in graph theory parameters are selected as brain network features. By analyzing the calculation principles of the conventional convolutional layer and the depth separable convolution unit, the computational complexity of them is compared. The depth separable convolution unit decomposes the traditional convolution process into spatial convolution for feature extraction and point convolution for feature combination, which greatly reduces the number of multiplication and addition operations in the convolution process, while still being able to obtain comparisons. Aiming at the special convolution structure of the depth separable convolution unit, this paper proposes a channel pruning method based on the convolution structure and explains its pruning process. Multimodal neuroimaging can provide complete information for the quantification of Alzheimer’s disease. This paper proposes a cascaded three-dimensional neural network framework based on single-modal and multi-modal images, using MRI and PET images to distinguish AD and MCI from normal samples. Multiple three-dimensional CNN networks are used to extract recognizable information in local image blocks. The high-level two-dimensional CNN network fuses multi-modal features and selects the features of discriminative regions to perform quantitative predictions on samples. The algorithm proposed in this paper can automatically extract and fuse the features of multi-modality and multi-regions layer by layer, and the visual analysis results show that the abnormally changed regions affected by Alzheimer’s disease provide important information for clinical quantification.

## Introduction

Alzheimer disease (AD) is a neurodegenerative disease in the brain. It is one of the most common types of dementia, accounting for about 60–80% of the total number of dementia patients (Lee et al., 2019; Spasov et al., 2019). AD usually has a chronic or progressive nature, and a variety of high-order cortical dysfunctions appear, manifested by symptoms such as cognitive decline, reduced judgment and memory loss, and ultimately lead to the loss of independent living ability. Many AD patients need to rely on the help of others to maintain a normal life, which brings a huge burden to caregivers (Martinez-Murcia et al., 2019; Noor et al., 2020). These burdens include various pressures on social life, psychological aspects, physical activities, and economic levels. AD has become a common problem faced by the whole world. To overcome the pathogenesis of AD, it is urgent to carry out early quantification and treatment. Mild Cognitive Impairment (MCI) is the early stage of AD. The annual conversion rate of MCI to AD is as high as 10–15%, while the annual conversion rate of healthy people to AD is only 1–2% (Ju et al., 2017). Certain cognitive training and rehabilitation can delay the development of AD, and some can return to normal. However, once it enters the AD stage, no effective therapeutic drugs have been developed clinically, and this process is irreversible (Bi et al., 2020a; El-Sappagh et al., 2020).

In recent years, the research on the combination of artificial intelligence technology and medical big data has achieved a lot of research success in the medical field (Li et al., 2019). Artificial intelligence technology can help doctors and patients predict more potential diseases. At the same time, medical big data also provides a good foundation and platform for the application of artificial intelligence technology. Among them, deep learning is a member of the artificial intelligence army. The core concept of deep learning is to simulate the multi-level information processing method of the brain, and interpret the input information in layers to obtain a series of characteristics of the input information (Luo et al., 2017; Shi et al., 2017). In the medical field, deep learning can not only perform image analysis and intelligent quantification of diseases, but also improve the efficiency of medical data collection and processing, thereby improving the accuracy of doctors in diagnosis and treatment of diseases, so that patients can get more timely, more complete, and more accurate treatment (Chen et al., 2019).

In actual clinical quantification, doctors often need to analyze multiple modalities of image data, and integrate multiple quantitative information, combined with experience knowledge, in order to make an objective judgment on the patient’s condition. This paper uses graph theory to extract brain network features and verify the effectiveness of the features. In this paper, two kinds of brain networks are established, and three graph theory parameters of node efficiency, node degree, and node betweenness centrality are extracted respectively. Through significant difference analysis, graph theory parameters with obvious differences between normal people and AD patients are taken as brain network characteristics. Specifically, the technical contributions of this article can be summarized as follows:

First: This article analyzes the computational complexity in the convolutional layer, introduces several special convolution structures, and focuses on the depth separable convolution structure, and based on the convolution unit, a new channel pruning is developed. We elaborated the channel pruning process for a single convolution unit, analyzed the compression effect of this method on the convolution unit, and then introduced the pruning process for the entire network. The key issues of channel selection, pruning ratio selection and model performance recovery in the overall pruning process are discussed. The channel selection was carried out according to the APo Z channel importance evaluation criteria, and the channel pruning was carried out on Mobile Net.

Second: We try to use multi-modal brain imaging data, such as MRI images. Through non-invasive imaging technology, clear tissue structure of the patient’s brain can be obtained, PET images and changes in the function of various brain tissues can be obtained through changes in glucose metabolism. We combine the two modal data for comprehensive analysis, which can improve the specificity and sensitivity of AD and MCI quantification, effectively prevent misdiagnosis and missed diagnosis, and improve the credibility of computer-aided quantification.

Third: In this paper, 3D-CNNs are used to extract the features of local 3D image blocks, and the feature output of the intermediate convolutional layer corresponding to the MRI and PET images at the same location is taken as the input of the feature fusion network, and the local features at different locations are stitched together, and then we use the trained network to make the final quantification of the sample. In the experiment based on multi-modal data, the quantization accuracy of AD and NC based on cascaded 3D-CNNs reached the ideal level.

The rest of this article is organized as follows. Section 2 discusses related work. Section 3 carried out the extraction and analysis of the parameter features of Alzheimer’s disease brain network graph theory. Section 4 designs a channel pruning algorithm based on efficient convolution unit. Section 5 presents the results and analysis of cognitive function quantitative experiments. Section 6 summarizes the full text.

## Related Work

AD is essentially a disease that continues to deteriorate and is incurable, and the direct cause is still unknown. The main factors that cause Alzheimer’s are genetic, neurotransmitter, immune and environmental factors (Lu et al., 2018a; Jain et al., 2019; Raza et al., 2019). Although the pathological changes of AD begin to appear very early, its typical clinical symptoms do not show up until later. According to estimates by the Alzheimer’s Association, if there is no major breakthrough in AD prevention, by 2050, the number of AD patients worldwide is expected to exceed 155 million (Zeng et al., 2018). Studies have found that about 12% of MCI patients convert to AD every year, while the annual probability of converting to AD for elderly people with normal cognitive function is only 1–2% (Oh et al., 2019). The high conversion rate of MCI to AD on the one hand shows that MCI is a high-risk group of AD, on the other hand, it also reflects the importance of early quantification of AD (Bi et al., 2020b). Therefore, if the patients with MCI can be screened out based on the early quantitative examination reports of the patients and their disease development can be predicted, preventive intervention can be carried out on the patients and standardized drug treatments can be given to the patients, thereby preventing or delaying the occurrence of AD (Choi et al., 2018; Lu et al., 2018b).

When doctors are faced with huge medical pathology data, the workload of quantifying AD is too complicated and there are some subjective predictions. For example, MRI images require doctors to go through the naked eye, linear measurement, area measurement, volume measurement, and MRI value measurement. Therefore, the accuracy and efficiency of AD quantification results may be further improved. At present, most researches use simple data analysis techniques and machine learning techniques to make quantitative predictions on MRI images, and on the other hand, researches are based on a single AD neuroimaging data, molecular biology data or genetic examination data. The use of deep learning technology to analyze MRI image performance text reports and multiple AD clinical examination data has not been studied in depth. In response to the above problems, related scholars proposed a new quantitative model and predictive model based on deep learning, designed and implemented an Alzheimer’s assisted quantitative medical system, which separately reported on MRI images. And multiple AD clinical examination data quantification can assist doctors in quantifying and predicting the development of the patient’s condition, providing more treatment time for preventing or delaying AD.

The quantification of Alzheimer’s disease needs to be combined with the patient’s medical history, family history, neuropsychological evaluation and other examinations, and the cause of the quantification needs to be based on clinical manifestations, biomarkers and structural images (Tang et al., 2019). Nowadays, domestic and foreign researches on quantitative prediction methods for Alzheimer’s disease are mostly based on MRI images in structured images, and then use different research methods for research, as well as using biomarkers and neuropsychological examinations (Wang et al., 2019).

Structured images include Computed Tomography (CT) and Magnetic Resonance Imaging (MRI). The early lesions of AD mainly involve the hippocampus, and CT is difficult to accurately display the structure of the hippocampus. Therefore, the role of CT in distinguishing and quantifying AD is limited. MRI shows that the abnormal changes of dementia are more sensitive than CT, and is recognized as the best imaging method to quantify and display the morphological abnormalities of dementia (Li et al., 2018). Therefore, MRI is more popular in quantitative research on the development of AD. Relevant scholars have investigated the gray matter and differences in MRI images, using posture morphology and statistical analysis methods to achieve the quantification of normal, mild cognitive impairment, and Alzheimer’s disease (Zeng et al., 2019). Based on Adaboost, the researchers analyzed the hippocampus volume in MRI images and studied the significant differences in the low-frequency amplitude of the three brain regions of normal, mild cognitive impairment, and Alzheimer’s, so as to quantify the condition of Alzheimer’s disease (Choi et al., 2019). Relevant scholars use principal component analysis to reduce the dimensionality of MRI images, and then use linear regression models to predict the development trend of the MMSE scale for patients with Alzheimer’s disease in one year (Gulhare et al., 2017; Vu et al., 2018). However, principal component analysis is used for dimensional reduction techniques. The disadvantage is that the interpretability of the established model is poor.

The method based on the prior knowledge area is based on the prior knowledge obtained by researching AD histological or imaging data. Generally, the features of some important regions can express information with rich discriminative power for AD, and these features can be extracted for quantification. The hippocampus is located in the medial temporal lobe and is one of the few areas where severe structural changes occur in AD (Mazurowski et al., 2019). Therefore, the geometrical characteristics of the hippocampus are often used as effective biomarkers. Research by related scholars has shown that hippocampal texture is better than volume shrinkage measurement in terms of predicting the conversion from MCI to AD based on SVM (Kumar et al., 2017). In fact, the effects of AD also manifest in other brain regions. Related scholars use the shape differential morphology measurements of the left and right amygdala, hippocampus, thalamus, caudate nucleus, putamen, globus pallidus, and lateral ventricle as features, and use LDA to predict AD transformation (Zhang et al., 2019).

The rapid development of deep learning has become a good supplement to traditional machine learning algorithms, and has also provided new means for the quantification and prediction of various neurodegenerative diseases, and has been increasingly used in the field of neuroimaging (Kam et al., 2019). However, deep learning algorithms still have certain limitations in neuroimaging research. The existing quantification methods of AD, MCI, and HC based on deep learning have generally achieved high accuracy in the quantification of AD group vs. HC group, but the accuracy rate is slightly less in AD group vs. MCI group and MCI group vs. HC group. This is due to the large differences in the imaging data of AD and NC patients. In the current quantification of AD, MCI and HC by researchers, the accuracy rate is relatively low. On the one hand, although deep learning models usually do not require pre-defined features and can search for and discover complex structural features that characterize the problem based on data, the feature extraction and analysis technology of neuroimaging is currently relatively mature and will be based on neuroimaging preprocessing (Vieira et al., 2017; Le et al., 2019). The extracted features are used to construct a deep learning model, which is very likely to accelerate the convergence speed of model training, reduce the demand for training data, and improve model performance (Liu et al., 2018). On the other hand, the large data training samples required for deep learning and the large number of parameters required for model adjustment can easily lead to a much higher computational complexity and the amount of data required for training models than traditional machine learning models (Jha and Kwon, 2017; Huang et al., 2020; Wingate et al., 2020). Therefore, although current neuroimaging databases have been well developed, their scale may limit the performance of deep learning to a certain extent. Therefore, this article uses traditional machine learning methods to analyze and study the quantitative problems of AD, MCI, and HC.

## Alzheimer’s Disease Parameter Feature Extraction

### Multimodal MRI Image Preprocessing

**FIGURE 1:**
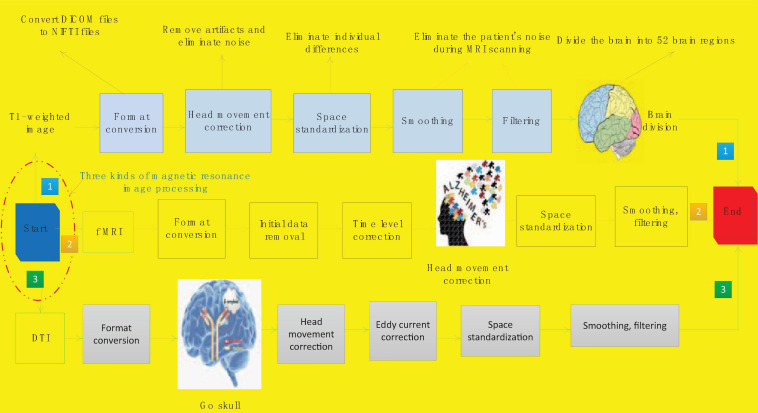
The original image preprocessing process of Alzheimer’s disease.

The scanned magnetic resonance images are all images output by the machine. These images have noise and the format is not convenient for computer processing. In order to facilitate the further processing and research of the image, it is necessary to perform the image preprocessing process first. There are many steps in the preprocessing process of the three modalities of magnetic resonance images are similar. We will introduce the preprocessing process of the different modalities one by one. Figure 1 shows the flow of magnetic resonance image processing for Alzheimer’s disease.

First we complete the preprocessing process of T1 weighted image. The first step is format conversion, which converts DICOM files into NIFTI files. The format of the original MRI image is generally DICOM format. DICOM is the unified output format of medical imaging machines. The images scanned in this format are independent images and need to be read with specific software, which is very inconvenient. It is not conducive to continue processing research. NIFTI file is an advanced medical image storage format, which has many advantages, such as convenient calculation and analysis, strong format universality, and centralized collection of corpus. We can continue processing the converted data.

The second step is head movement correction. When a patient is scanning for magnetic resonance imaging, it is inevitable that there will be some slight jitter, which will cause noise and artifacts in the collected images. In order to remove artifacts and noise, it is necessary to perform head movement correction on the scanned T1-weighted image. Spatial standardization is an important step to eliminate individual differences. Due to differences in the patient’s head volume, shape, etc., all images are mapped once and mapped to the MNI standard space. This will eliminate the influence of patient individual differences on subsequent experiments and make the results more accurate. The fourth step of smoothing filtering is also mainly to eliminate the noise of the patient during the scanning magnetic resonance.

The last step is the division of brain regions. The brain division of T1-weighted image is not actually a routine preprocessing step. In this study, the T1 weighted image is mainly used as a template for the construction of the brain network using f MRI and DTI data. So here is included in the preprocessing process. This process will use the brain division method to divide the brain into 52 brain regions. The preprocessing process of T1 weighted image is now completed.

The f MRI preprocessing process is implemented on the MATLAB open-source toolkit GRETNA (Graph Theoretical Network Analysis Toolbox). The second preprocessing step of f MRI is to remove the initial data. The initial 5 data of f MRI are unstable because of the unstable blood oxygen signal, so the data obtained by f MRI scan is not reliable and needs to be removed. The third step of preprocessing is time level correction. f MRI scans the odd-numbered slices first and then the even-numbered slices during the scanning process. The scanning time of adjacent slices is very different, and the processing can be continued after time correction.

The process of DTI preprocessing is done on MATLAB’s open source toolkit PANDA (Pipeline for Analyzing Brain Diffusion Images). There are only two differences between DTI preprocessing and T1 weighted image preprocessing. The second step of pre-processing is to remove the skull. DTI is a structural image, and the structure of the skull is more obvious. In order to prevent the skull from interfering with the research in the subsequent research process, it was removed in the preprocessing process. The fourth step of pretreatment process is eddy current correction. Due to the frequent switching of the dispersion gradient, the image will produce eddy current distortion. The eddy current correction can remove the eddy current distortion and make the imaging result more accurate. So far, the preprocessing of f MRI, DTI, and T1-weighted images is all over.

### Brain Function Connection Network Acquisition

Brain functional connection is obtained from f MRI data, which can reflect the synchronization of functional activities between brain regions by calculating the correlation of time series signals between brain regions. The network formed by the functional connections between all brain regions of the whole brain is called Functional Connectivity Network (FCN).

Through the preprocessing process, the text has obtained the brain template. In this way, the nodes of the brain network can be defined smoothly. In the process of defining the brain function to connect the network edge, the text is defined by the correlation of the mean value of all voxels between the two brain regions. The formula for calculating the time series correlation coefficient between any two brain regions is as follows:

(1) $$c_{a,b}\left(u\right)=\frac{Cov_{a,b}\left(u\right)}{\sqrt{var\left(a\right)\cdot var\left(b\right)}}$$

In the above formula, var(a) and var(b) respectively represent the variance of the mean value of all voxel time series in the two brain regions over time, and Cov a b(u) represents the covariance of the time series mean value of the two brain regions. According to the functional magnetic resonance imaging data, the time series of each voxel can be obtained, and the calculation of the time series correlation coefficient between any brain regions can be completed through mathematical calculation.

**FIGURE 2:**
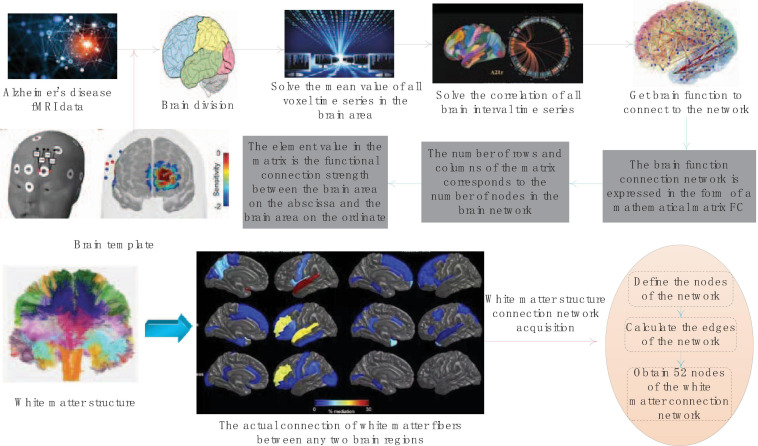
Schematic diagram of brain function connection network acquisition.

The obtained correlation coefficient between brain regions is the strength of the functional connection, but only when the correlation coefficient between the two brain regions exceeds the threshold, can it be determined that there is a functional connection between the two brain regions. The larger the correlation coefficient value, the higher the even strength. The side of the brain function connection network is the above-mentioned functional connection strength. The functional connection strength between any two brain areas of the divided 52 brain areas is calculated, and the acquisition of the brain function connection network is completed. The schematic diagram of the acquisition of the entire brain function connection network is shown in Figure 2.

The brain function connection network will be expressed in the form of a mathematical matrix FC. The number of rows and columns of the matrix corresponds to the number of nodes in the brain network. The element values in the matrix represent the magnitude of the functional connection strength between the abscissa brain area and the ordinate brain area. All elements together constitute the weight of the brain function connection network edge.

The white matter structure connection measures the connection of white matter fibers that actually exist between two brain regions. The network formed by the connection of white matter fibers between all brain regions of the whole brain is called DTI Structural Connectivity Network (DTISCN). The white matter structure connection network acquisition is similar to the process of f MRI data acquisition FCN. It also defines the nodes of the network first, and then calculates the edges of the network. The process of defining nodes in the white matter structure connection network is the same as that of FCN. The two imaging methods of the same sample use exactly the same templates, so the white matter structure connection network also has 52 nodes.

The white matter structure connection network will use the anisotropy value FA obtained from DTI data to define the white matter fiber connections between brain regions. The physical meaning of FA value indicates the strength of dispersion, and the number and density of white matter fibers can be reflected by the size of FA value. The method of solving the FA value of each voxel is as follows:

(2) $$FA=\frac{\sqrt{3\cdot \left[\left(\eta_{1}-\eta^{2}\right)+\left(\eta_{2}-\eta^{2}\right)+\left(\eta_{3}-\eta^{2}\right)\right]}}{\sqrt{2\cdot \left(\eta_{1}^{2}+\eta_{2}^{2}+\eta_{3}^{2}\right)}}$$

Different values of η indicate the degree of dispersion of each voxel in different directions, and η’ indicates the average value of the degree of dispersion in different directions. The FA value between any two brain regions can be obtained from the FA value of each voxel of these two brain regions. However, not all the FA values of the brain regions are reasonable and effective, because some FA values cannot describe the white matter fiber connections in the brain regions. Here we will introduce a probabilistic fiber tracking algorithm, which can eliminate invalid FA values and leave valid FA values. The probabilistic neural tracking algorithm calculates the relationship between the gradient information and the anisotropic FA. The threshold of FA is 0.2. If the anisotropy value is lower than 0.2, it is assumed that there are no nerve fibers in the voxel or that nerve fiber disconnection occurs. The threshold of the angle is set to 35°. If the angle exceeds 35°, it is considered the intersection of two nerve fibers instead of one nerve fiber.

In this paper, the white matter structure connection network is obtained with the assistance of MATLAB toolkit PANDA. We can obtain the nodes and edges of the white matter structure connecting network. When the FA value is still confirmed after the fiber tracking between the two brain regions is completed, it is assumed that there is a white matter fiber connection between the two brain regions. The larger the FA value, the stronger the white matter fiber connection strength. Similar to FCN, DTISCN will be obtained by the anisotropic FA matrix between brain regions. The FA matrix can also be visualized for intuitive observation. Similar to the FC matrix, each point of the FA matrix represents the strength of the white matter fiber connection between the abscissa brain area and the ordinate brain area. Because the fiber connection has no directionality, the matrix is a symmetric matrix. Through the visualized graph, it can be seen that the FA value does not exist in many places. One part of the FA value does not exist originally, and the other part is that the FA value is removed after the fiber tracking is completed. The FA matrix can be used to extract the connection network characteristics of the white matter structure.

### Extraction of Parameter Features

Graph theory parameters include node degree, node efficiency, and node betweenness centrality. The calculation methods and physical meanings of different parameters are different. The node degree is a description of the importance of a node in the network, specifically expressed as the sum of the weights of all other node edges in the network that have a connection relationship with the node. The larger the node degree, the closer the connection between the node and other nodes, and it also reflects the high importance of the node in the entire network. The calculation formula of node degree of i-node is as follows:

(3) $$D_{i}=\sum_{j\to N}w_{ij}$$

In the above formula, *w*_*ij*_ represents the weight value of the edge between node *i* and node *j*, and *N* represents the node in the network. The value in the connection matrix FC reflects the weight of the brain function connecting the network edge. The anisotropy matrix FA reflects the weight of the white matter structure connected to the network edge. In this study, changes in the degree of nodes can reflect changes in the importance of corresponding brain regions. By comparing normal people with AD patients, changes in the importance of brain regions in AD patients can be found.

Node efficiency, also known as local efficiency, mainly reflects the efficiency of the information transfer process between the node and surrounding nodes. This indicator can not only reflect the efficiency of information flow between the neighbors of the node, but also reflect the degree of optimization of the local network. Therefore, the node efficiency can be expressed as the average of the sum of the reciprocal of the shortest path of each node in the network composed of the node’s neighborhood. The calculation formula for node efficiency of i-node is as follows:

(4) $$E_{i}=\sum_{j\neq k}\frac{1}{L_{jk}}\frac{1}{N_{G_{i}}\left(N_{G_{i}}-1\right)}$$

In the above formula, *G*_*i*_ is a network formed by the neighborhood of node *i*, and *j* and *k* are two nodes in the neighborhood network. *L*_*jk*_ is the shortest path between any two nodes in the neighborhood network. According to the FA matrix and the FC matrix, the node efficiency of each node in the two networks of FCN and DTISCN can be calculated respectively. Node efficiency can reflect the efficiency of brain processing information and the ability to resist attacks. The decline in the efficiency of brain nodes will reflect the damage to the brain by diseases.

Node betweenness centrality is also an index used to describe the role and status of a node, which is different from the perspective of node degree description. Betweenness centrality describes the criticality of nodes in the process of network information processing from the perspective of information flow. Node betweenness centrality is defined as the ratio of the number of paths containing the node among all the shortest paths in the entire network to the number of all shortest paths in the entire network. If the betweenness centrality of nodes changes, it means that the shortest path of the whole brain network will change, and the efficiency of the entire network will also change. The formula for calculating betweenness centrality of i-node is as follows:

(5) $$B_{i}=\sum_{i\neq j\neq k}\frac{\sigma_{jk}\left(i-1\right)}{\sigma_{jk}}$$

Node betweenness centrality can also be obtained through FA and FC matrices. Betweenness centrality is a very useful indicator for brain networks, it reflects the size of the brain area in processing information. The change of betweenness centrality in AD patients can reflect the damage and involvement of the brain area.

Graph theory parameters are obtained above, and each graph theory parameter is a characteristic component. But these graph theory parameters can only be used as feature components, and only feature components with significant differences can be called features. Therefore, this article will verify the differences of graph theory parameters, and select the feature components with significant differences as the features of FCN and DTISCN.

In the DTISCN features, the left side of the basal forebrain and the right side of the basal forebrain showed changes in graph theory parameters. It can be inferred that the structure of the basal forebrain has been damaged. Of course, there are many features that are also corroborated with the symptoms of AD patients. I cannot explain them one by one here, but it is certain that there are some abnormalities in the brain function network and white matter structure connection network of AD patients. At the same time, these features are reasonable and effective.

## Channel Pruning Algorithm

### Complexity of Convolutional Neural Network

In deep neural networks, complexity is divided into computational complexity and space complexity, which have the following effects on the network:

(1) Computational complexity
  The training and prediction speed of the model is determined by the computational complexity. The higher the computational complexity, the more time it takes for the forward calculation of the model. The model cannot be tested quickly and cannot be applied in scenarios with high real-time requirements. At the same time, the higher the computational complexity, the more time it takes to train the model. The longer it is, the more it is impossible to verify and improve the model in time.
(2) Space complexity
  The number of parameters in the model determines the space complexity of the model. Generally speaking, a larger model has more parameters, and the better the fitting ability of the model. Then the larger the model, the more data is needed to perform the model. The data set in real life is usually not too large, which makes the model more prone to overfitting.

The main operation layer in the convolutional neural network is the convolution layer, and the calculation principle of the convolution layer is as follows:

(6) $$S\left(i,j\right)=\sum_{m}\sum_{n}K\left(i-m-1,j-n-1\right)\cdot I\left(m,n\right)$$

Suppose *I* is the input layer, *K* is the convolution kernel, the size of *I* is D1 × D1 × M, D1 is the width and height of the input feature map, M is the dimension of the input feature map, and the convolution kernel size is D2 × D2 × M × N, I get an output feature map with a dimension of D1 × D1 × N through the convolution kernel, so the computational complexity is O(D1 × D1 × D2 × D2 × M × N), and the convolutional layer space complexity is O (D2 × D2 × M × N), the total computational complexity and space complexity of the convolutional neural network is the sum of the computational complexity and space complexity of each convolutional layer.

Some convolution kernels in Alex Net use a size of 5 × 5 or even 7 × 7, which greatly increases the complexity of the network. Starting from the VGG neural network, convolutional neural networks have generally adopted 3 × 3 convolution kernels to obtain features. When cutting the model, since the spatial size of the convolution kernel (3 × 3) is already small, the cutting of the network model usually starts with the number of channels, that is, reducing the value of M or N.

Inception_V1 constructs four parallel convolution/pooling modules of different sizes in an Inception structure, which effectively increases the width of the network, but doing so also causes a surge in the time and space complexity of the network.

**FIGURE 3:**
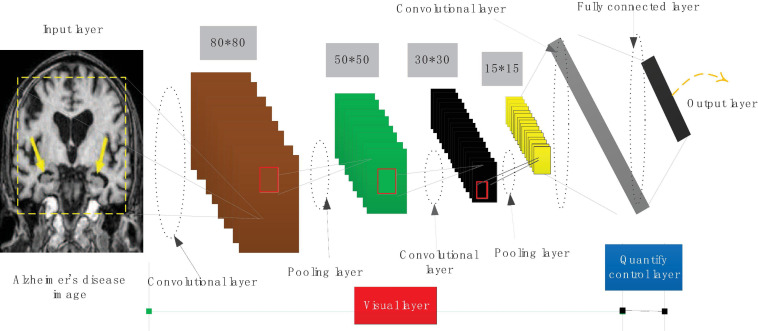
Convolutional neural network structure.

The Inception module first uses 1 × 1 convolution to reduce the dimensionality of the input feature map, and performs a weighted feature combination on the features of different channels, and then uses 3 × 3 convolution to simultaneously map the spatial dimension and the channel dimension. It first uses 1 × 1 convolution on the channel correlation to map the input feature map to a space with several dimensions smaller than itself, which is equivalent to multiplying each channel map by a different factor to make a linear combination, using 3 × 3 Convolve these small spaces, and map its space and channel correlation at the same time. The convolutional neural network structure is shown in Figure 3.

Using 1 × 1 convolution to reduce dimensionality can reduce the computational complexity by more than 3 times. According to the two-dimensional convolution input and output size relationship, for the same input size, the output of a single 5 × 5 convolution is exactly the same as the output of two 3 × 3 convolution cascades, that is, the receptive field is the same.

Also according to the complexity analysis formula, this replacement can effectively reduce the space and time complexity. Using this convolution structure can use the saved complexity to increase the depth and width of the model, so that the complexity of the model remains unchanged.

### Depth Separable Convolution and Pruning

**FIGURE 4:**
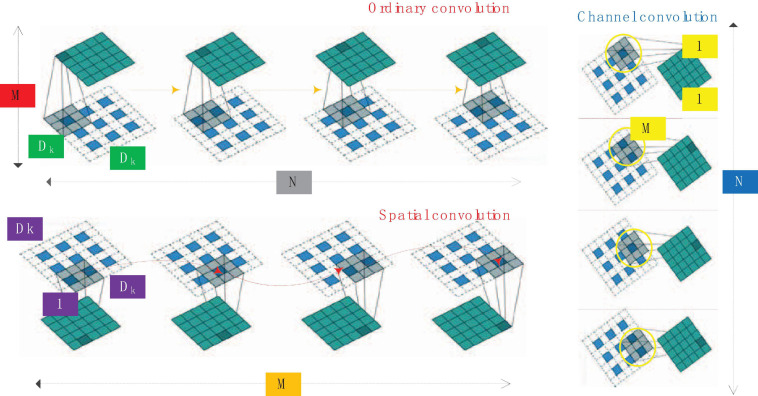
Comparison of ordinary convolution, spatial convolution and channel convolution.

For convolution, the convolution kernel can be regarded as a three-dimensional filter: channel dimension + spatial dimension (corresponding to the width and height of the feature map). The conventional convolution operation is actually to realize the combination of channel correlation and spatial correlation. There is an assumption behind the Inception module: the combination of spatial features of the convolutional layer and the combination of channel features can be performed separately, and better results can be achieved by doing it separately. Depth separable convolution solves traditional convolution into deep convolution and channel convolution. The comparison of ordinary convolution, spatial convolution and channel convolution is shown in Figure 4.

The 1 × 1 convolution method of the deep separable convolution structure greatly reduces the amount of calculation in the forward operation process. Not only that, in Mobile Net, about 95% of the multiplication and addition operations come from 1 × 1 convolution (accounting for parameter 95% of the number), the large use of 1 × 1 convolution means that it can be directly implemented using highly optimized matrix multiplication algorithms (such as GEMM), which greatly improves computational efficiency. The method of network pruning has been widely used in convolutional neural network model compression. In early work, network pruning was considered as an effective method to reduce network complexity and reduce overfitting. Pruning the model with the best performance so far reduces the network complexity without loss of accuracy. Generally speaking, the following methods are used for network pruning:

(1) Use ordinary methods to train a complete convolutional neural network. (2) Sort the weight of each layer according to the absolute value of its weight. (3) Select the weight whose absolute value is lower than a certain threshold and remove it. (4) Retrain the network after pruning to achieve the performance before pruning as much as possible.

Usually this method can greatly reduce the number of network parameters in the fully connected layer. The method of network weight pruning can often greatly reduce the amount of network parameters, but weight pruning often has the following disadvantages.

(1) These network pruning methods are only for the fully connected layer. The fully connected layer is often the part with the most redundant parameters. In practical applications, we often abandon the fully connected layer and replace the corresponding part with the average pooling layer. Therefore, in the existing convolutional neural networks, the convolutional layer often accounts for most of the calculation and is the most time-consuming part. In general, the above algorithms can achieve faster speeds or less storage capacity, but they rarely achieve significant acceleration while compressing the entire network.
(2) Weight pruning is easy to produce sparse connections, and the computational efficiency of sparse neural network structure is not as good as that of ordinary tightly connected neural networks.

Channel pruning is another weighted pruning method. Unlike the pruning method that removes a single neuron connection in neuron pruning, channel pruning removes less important channels in the entire convolutional layer. Each filter corresponds to a channel of the activation layer, and the expressive ability of each channel filter is closely related to its corresponding activation layer. A simple strategy to calculate the expression ability of the filter is to calculate the average percentage of zero activation value (Average Percentage of Zeros, APo Z) for each channel of the activation layer. The higher the APo Z, the lower the importance of the filter, and the lower it should be removed after a certain threshold.

### Channel Convolution

Currently, for the problem of network pruning, there is no suitable benchmark network architecture as a criterion for judging the performance of pruning. At present, the most commonly used convolutional neural network structures in network pruning, such as Alex Net, Goog Le Net, Res Net, etc., these models are effective in image quantization tasks, but they are in order to achieve the best performance and performance in the Image Net competition. The extreme accuracy rate increases, and the design parameters are seriously excessive, so these convolutional neural networks can easily obtain extremely large multiples of compression. Therefore, these methods can often only prove that a certain method is correct, but it is of little significance. The more meaningful challenge is to compress those models that are inherently more efficient in terms of speed and accuracy trade-offs. This article tries to simplify the Mobile Net itself. The channel pruning operation is performed on the network. The main structure in Mobile Net is a depth separable convolution unit, so this paper proposes a channel pruning method based on a depth separable convolution unit.

**FIGURE 5:**
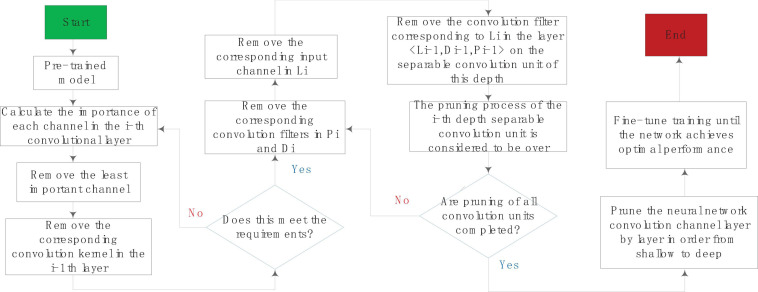
The overall process of channel pruning.

(1) Pruning process
  The depth separable convolution unit is composed of multiple 3 × 3 spatial convolution layers and multiple 1 × 1 channel convolution layers. The main calculation amount of this unit is concentrated in the 1 × 1 channel convolution. If the pruning operation is performed on the channels obtained by the 3 × 3 spatial convolution layer, the number of input channels of the 1 × 1 convolution layer can be significantly reduced, thereby reducing the computational complexity of the 1 × 1 channel convolution.
  Now we consider explaining this method from another angle. In the depth separable convolution, the 3 × 3 spatial convolution can be regarded as the feature extraction process, and the features of each channel of the feature layer are filtered to obtain the features. After the features are obtained, the features are performed by the 1 × 1 channel convolution method. The introduction of channel pruning after the spatial convolutional layer can be regarded as introducing a feature selection process to retain the more important features in image quantization, detection or segmentation tasks, and filter out the less important features.
  We use a triple < Li,Di,Pi > to represent the i-th depth separable unit, Li refers to the input unit, Di refers to the spatial convolution in the depth separable convolution structure, and Pi refers to the channel convolution in the structure, namely convolution part. The activation layer in the figure refers to the activation layer after the Di layer, which is the input unit of channel convolution. The goal of channel pruning is to cut the less important channels in the activation layer. At the same time, the corresponding convolutional layers in Di and Pi will also be removed.
  Each filter in Di corresponds to a channel in the activation layer, and the importance of the feature channel is often evaluated by some index. The light-colored layer in the activation layer represents the output channel with lower importance and should be removed. The corresponding convolution filter in Di is removed, and the convolution filter that uses this channel as input is removed at the same time, and the output dimension of the depth separable convolution unit remains unchanged. From the calculation principle of the depth separable convolution, we know that a certain channel j of the activation layer is obtained by convolution of the j-th channel of the input unit with the 3 × 3 filter of the j-channel of the spatial convolution layer. The channel is removed, which means that the j-th channel of the input layer should also be removed. For the convolution filter that should be removed, in the pruning process, the corresponding convolutional layer weight value is set to 0, and the corresponding learning rate is also set to 0. Therefore, the training and inference process of the convolutional neural network is not in calculations. After the network fine-tuning training is completed, the convolutional filter with an ownership value of 0 in the network will be removed. At this point, the unimportant convolutional layer in the neural network has been completely removed. The calculation amount of the entire module has been calculated as D1 × D1 × D2 × D2 × M + M × N × D1 × D1. Now some channels are removed by the method of channel pruning. It should be noted that the pruning process does not reduce the output dimension of the convolution unit, but only cuts off the less important feature layer and its corresponding weight. Therefore, assuming that M × ε channels (0 < ε < 1) are removed, the entire depth can be separated, the calculation amount of the convolution unit becomes D1 × D1 × D2 × D2 × M × (1−ε) + M × N × D1 × D1 × (1−ε), which is greatly reduced compared to the original convolutional neural network. And the output dimension size N will not change.
  The pruning of the i-th depth separable convolution unit will also affect the i-1th convolution unit. Assume that the j channel of the input layer Li of the i-th depth separable convolution unit is removed, and Li is generated by the channel convolution part Pi-1 of the i-1th depth separable convolution structure, so the corresponding 1 × 1 convolution part can also be removed. It can be seen that the channel pruning method based on the depth separable unit has better interpretability than the ordinary convolution unit, especially when dealing with multilayer structure pruning.
  A complete channel pruning process based on depth separable convolution unit is shown in Figure 5. For the i-th depth separable convolution unit < Li,Di,Pi >, all channels of the spatial convolution layer Di output are obtained according to a certain importance evaluation criterion degree of importance. Then you sort the convolution channels in the order of importance from small to large, remove some of the least important channels in the output, and then remove the corresponding convolution filters in Pi and Di and the corresponding ones in Li input channel. You remove the convolution filter corresponding to Li in the layer < Li-1,Di-1,Pi-1 > of the depth separable convolution unit, so that the pruning of the i-th depth separable convolution unit process is over. After the pruning of all convolutional units is completed, the network fine-tuning method is used to restore the lost performance of the model after pruning.

(2) Evaluation of channel importance and selection of pruning ratio
  The first and most important step of channel pruning is to evaluate the importance of feature channels. Important feature channels retain some of the more important features in the model. If removed, the accuracy of the model will be greatly affected. This article chooses APo Z as the evaluation standard for the importance of the convolution channel. In the depth separable convolution unit, the spatial 3 × 3 convolution layer is convolved separately for each input channel to obtain image features. Therefore, there is a one-to-one correspondence between the convolution channel and the output feature layer. After the feature of the spatial convolutional layer Di is taken, nonlinearity is introduced through the linear rectification activation function (Re Lu) to obtain the activation layer Ai. Therefore, the higher the APo Z in the activation layer, the greater the proportion of 0 in the activation layer, indicating the most positions of the feature layer are not activated, and their importance is low, and their corresponding spatial convolutional layer Di should be removed.

The Softmax function, also known as the normalized exponential function, is a generalization of the logical function, and the function form is as follows:

(7) $$\sigma {\left(z\right)}_{j}=\frac{e^{-zj}}{\sum_{k=1}^{K}e^{-zk}}$$

It can be seen from the formula that the Softmax function is actually the normalization of the discrete probability distribution of finite items, and Softmax is widely used in neural networks for multi-quantization problems.

It can be seen from the Softmax formula that the Softmax function is actually the gradient log normalization of the discrete probability distribution of finite items. Softmax is widely used in neural networks for multi-quantization problems. According to the Softmax function, the probability value corresponding to each soft label is obtained. In deep learning tasks, the goal of training is to optimize the loss function, and maximum likelihood estimation is generally used to construct the loss function in quantization problems. For the input x, assuming our class label is t, the goal of the quantization task is to find the most suitable model to maximize p(t| x). In the second quantification problem, we can know from the probability knowledge:

(8) $$p\left(t|x\right)={\left(1-y\right)}^{t-1}\cdot y^{t}$$

y = f(x) is the probability value predicted by the model, and t is the class label corresponding to the sample. Generalizing the two-quantization problem to a more general multi-quantization problem, p(t| x) is expressed as follows:

(9) $$p\left(t|x\right)=\prod_{i=0}^{C-1}p{\left(t_{i}|x\right)}^{t_{i}-1}=\prod_{i=0}^{C-1}y_{i}^{t_{i}-1}$$

In actual calculations, continuous multiplication may cause the final result to approach 0, and in the process of network back propagation, the exponential function is inconvenient to handle, so the likelihood function is generally taken as the negative logarithm of the logarithmic likelihood function to convert the problem of maximizing p(t| x) into a problem of minimizing the log-likelihood function.

(10) $$L\left(t,x\right)=-t\cdot \log p\left(t|x\right)=-\sum_{i=0}^{C-1}t_{i}\cdot \log y_{i}$$

Using the cross entropy function not only can measure the effect of the model very well, but also can easily calculate the derivative.

Now we need to select a data set, and get the output of each channel after activation of the spatial convolution layer Di for each image sample of the data set. We calculate the number of activation values of 0 in the feature map, divide it by the size of the feature map, and finally average all samples to get the APo Z value of the convolution channel corresponding to the spatial convolution layer. After that, we sort the feature channels in the order of APo Z from small to large, and remove the M × ε channel with the largest APo Z value according to a certain pruning ratio ε, and retain the convolution channel with the smaller APo Z value. The APo Z-based convolutional channel importance evaluation algorithm has the advantages of simple method and fast calculation, and is widely used in convolutional neural network channel pruning tasks.

According to APo Z, channels with low APo Z retain more effective information than channels with high APo Z. A spatial layer will output hundreds or thousands of characteristic channels. The more channels removed, the more effective the network performance, the greater the impact. There is currently no standard answer to this question. Different models and different data sets will have different pruning ratios.

The pruning of the upper layer of convolutional unit will have a certain impact on the next layer of convolutional layer unit, so there are two methods for the performance recovery after model pruning, one is the greedy pruning method, that is, the upper and lower layers are not considered as a result, all convolutional channels of the network are pruned at one time, and then the network is fine-tuned to restore network accuracy. Another way is to prun and fine-tune the neural network convolution channel layer by layer from shallow to deep until the network achieves the best performance. Both methods have their own advantages and disadvantages. The process of the greedy pruning algorithm is relatively simple, and the pruning steps and fine-tuning steps are time-consuming; the advantage of the layer-by-layer pruning method is that it is often more accurate.

## Cognitive Function Quantitative Experiment Analysis

### Experimental Data

**FIGURE 6:**
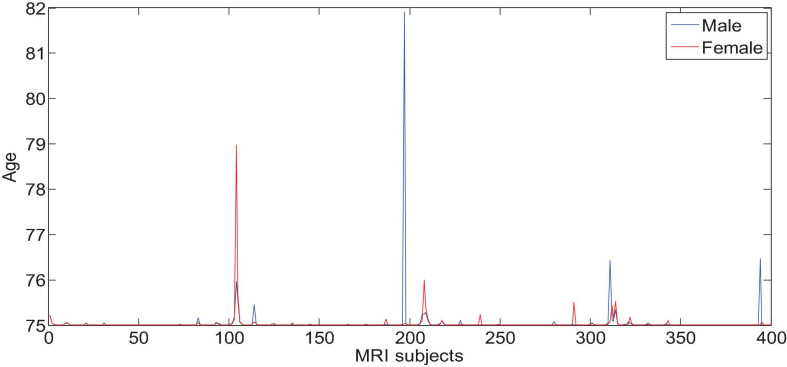
Age characteristics of Alzheimer’s disease of MRI subjects.

**FIGURE 7:**
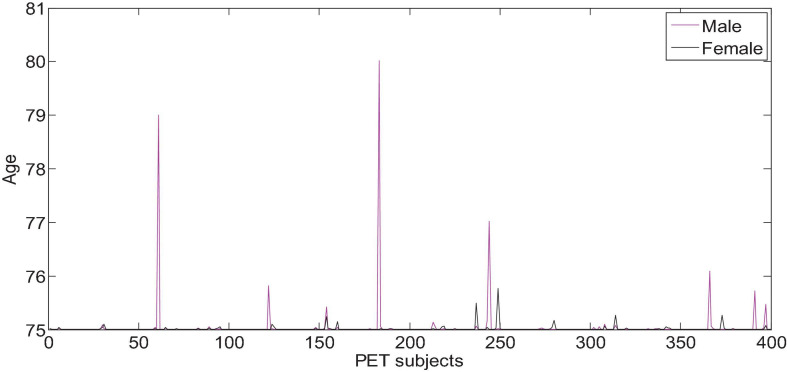
Age characteristics of Alzheimer’s disease of PET subjects.

All the data in the experiment comes from the ADNI database, and the age information of the subjects is shown in Figures 6, 7. We use all the initial moment data. There are a total of 800 subject samples containing MRI data, including 168 AD, 403 MCI and 229 NC, and a total of 339 multimodal data samples containing both MRI and PET, including 93 AD, 146 MCI and 100 NC.

The original image size is 256 × 256 × 256, and the surrounding pixels without information are removed after sampling by down 2. The maximum enclosing size of MRI and PET is 100 × 81 × 80. The cross-validation method is used to do ten-fold cross-validation. The training data set is enhanced by shifting sampling in all directions, which is increased by eight times. The verification data set and the test data set are not enhanced. We use the Adadelta gradient optimization algorithm to learn the weights of the network, and the batch size is set to 64. During training, the model tends to converge about 20 iterations. In this experiment, we will take several experiments to evaluate the model comprehensively on the three quantitative tasks of AD vs. NC, p MCI vs. NC and s MCI vs. NC. When training local 3D-CNN, the network weight of AD vs. NC is initialized in the same way as 2D-CNN, using Xavier for random initialization. Since the difference between mild cognitive impairment samples and NC is small, the transfer learning method is used to alleviate the problem of insufficient training caused by the small amount of training data. We will use the trained AD vs. NC network for the p MCI vs. NC network. The NC network is initialized. In the same way, the network of s MCI vs. NC is initialized with the network of p MCI vs. NC.

In order to more comprehensively verify the effectiveness of the cascaded neural network proposed in this paper, we launched a comparative experiment. It is worth noting that the following MRI monomodal experimental results are based on all the 800 MRI images at the initial moments. The PET monomodal and multimodal experiments used 339 recipients who participated in both MRI and PET scans. The following are the results and analysis of each experiment.

### Parallel 3D-CNNs Experiment

**FIGURE 8:**
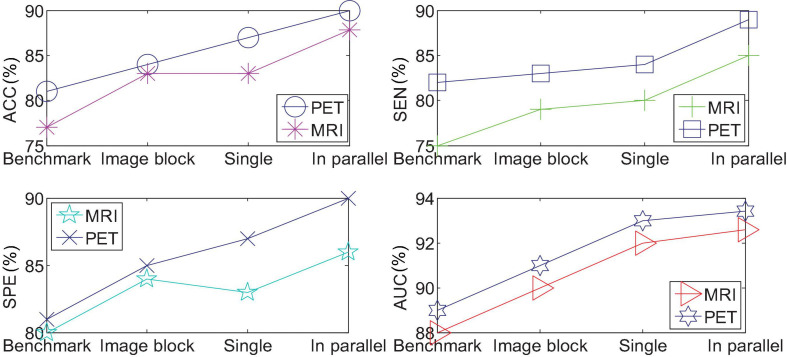
Comparison of parallel 3D-CNNs integration method and other methods.

We use single-modal images to quantify AD vs. NC, and compare the results of benchmark tests, single CNN quantification of the entire brain image, and single CNN quantification of the performance of partial images. Figure 8 shows the comparison results. Among them, the method used in “benchmark test” is to calculate Propensity Score Matching (p-score) based on two categories, obtain the probability estimation of each pixel, and use the verification set to select the first n pixels with the most discriminative ability, and then use the support vector machine to quantify the result. “A single image block with the best performance” represents the block with the highest quantization accuracy among the 27 image blocks. “Single CNN quantization” downsamples the entire brain image by 4 and inputs it to the same network as the quantized partial image block for quantization. “Parallel 3D-CNNs integration method” refers to the network structure.

From the experimental results in the observation table, it can be found that the 3D convolutional neural network model proposed in this article has a significant improvement in the effect of the benchmark test. The method of integrating 3D-CNNs can further improve the prediction of AD disease in a single modal 3D image. These evaluation indicators verify the effectiveness of the parallel multiple CNNs model proposed in this paper.

### Multimodal Fusion Experiment

We combined the local features of MRI and PET, and used the multi-modal data fusion method of cascaded multi-CNNs to quantify AD vs. NC, p MCI vs. NC and s MCI vs. NC, respectively, to verify the advantages of single-mode analysis.

**FIGURE 9:**
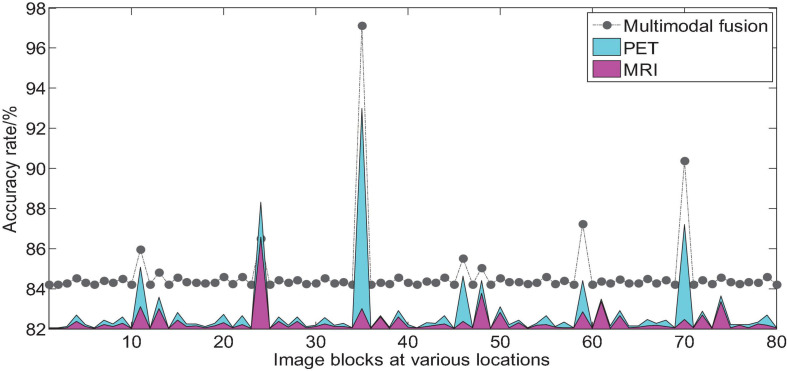
Comparison of AD vs. NC accuracy of 3D image blocks at various positions before and after multi-modal fusion.

We take AD vs. NC as an example to analyze the multi-modal fusion effect of a single location image block. The histogram shown in Figure 9 represents the accuracy results of the multi-modal fusion of 80 image blocks.

From the histogram shown in Figure 9, we can find that we use 2D-CNN to perform feature fusion of the local image blocks of the two modalities compared to the accuracy of the quantization of a single mode, which can improve the quantization performance in almost all positions. It can be seen that the multimodal fusion method proposed in this paper is effective.

**FIGURE 10:**
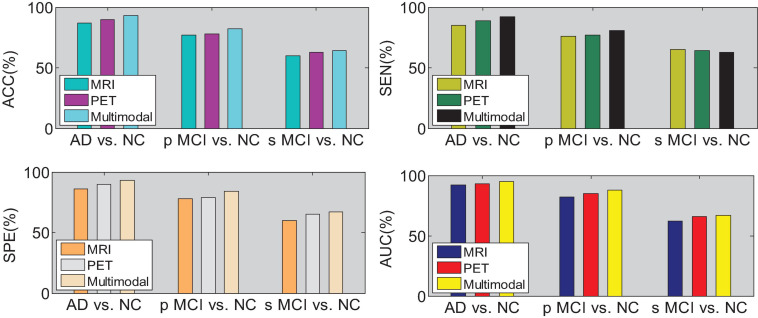
Performance comparison of single-mode and multi-mode under three quantitative tasks.

We then quantify the features of multiple local image blocks through a fully connected layer combination. Figure 10 shows the three quantization tasks of AD vs. NC, p MCI vs. NC and s MCI vs. NC in single mode and multi-mode. Among them, the results of “MRI” and “PET” in the figure are obtained directly from a single mode image. It can be seen from the results in Figure 10 that the quantified performance of the multi-modal data is greatly improved in all indicators compared with the single-modal data.

**FIGURE 11:**
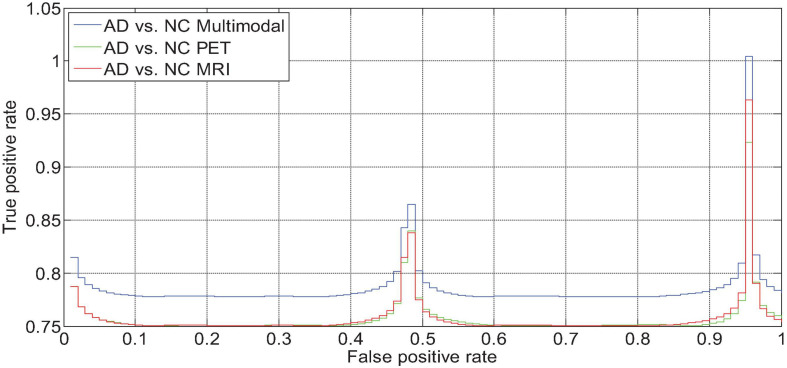
AD vs. NC fusion quantified ROC curve.

**FIGURE 12:**
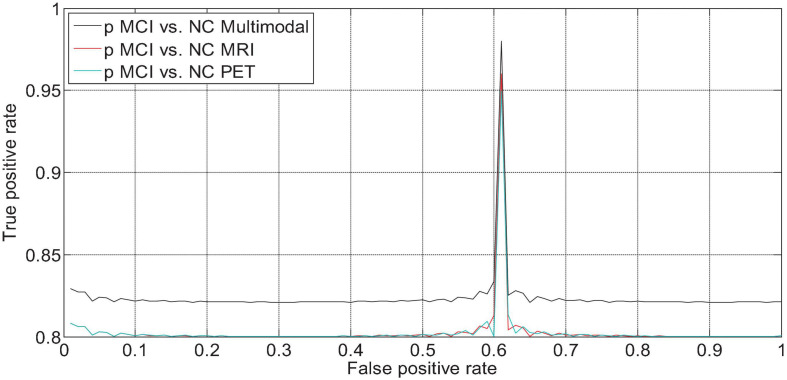
ROC curve of p MCI vs. NC fusion quantification.

**FIGURE 13:**
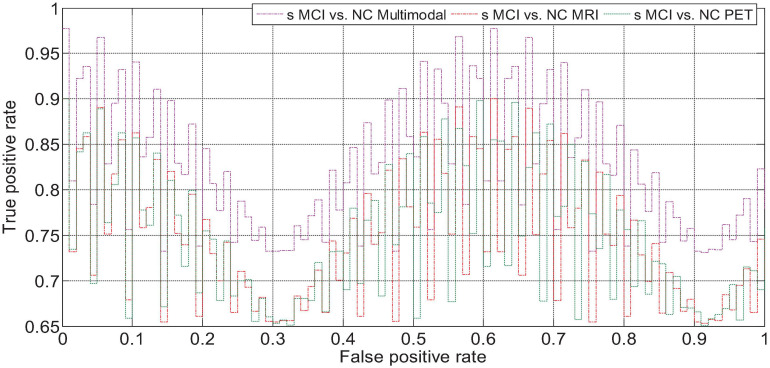
s MCI vs. NC fusion quantified ROC curve.

We drew the ROC curves of AD vs. NC, p MCI vs. NC, and s MCI vs. NC based on the network prediction results, as shown in Figures 11–13, respectively. Observing the ROC curve, it can be found that the performance of multimodal fusion is significantly better than that of single mode, and the performance of PET is slightly lower than the quantitative performance of MRI.

### Comparison With Other Fusion Algorithms

**FIGURE 14:**
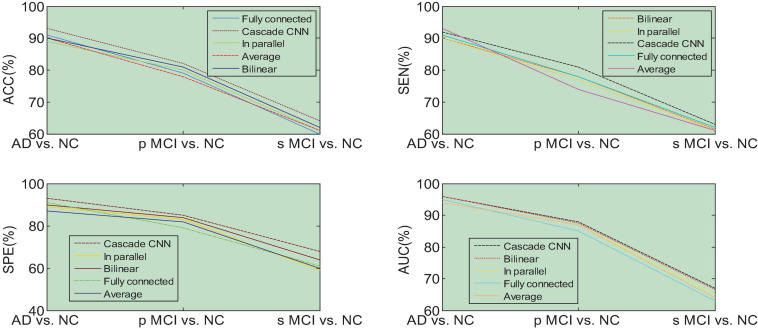
Performance comparison of five multi-modal fusion methods.

The features extracted based on the network are compared with the multi-modal fusion method with multiple feature fusion methods such as direct averaging method, feature stitching method, parallel method, and feature bilinear point multiplication. As shown in Figure 14, “average” refers to the average predicted probability of a single sample using two modalities as the final predicted probability of the sample, and “splicing” means concatenating the features of the fully connected layers of the two modalities into one. The dimensional features are re-quantified as the total features of the sample. “Parallel” means to connect the features of the last convolutional layer in parallel and then perform subsequent quantification. “Bilinear” means to use the features of the fully connected layers of MRI and PET for bilinear multiplication, which is fused into a feature matrix for processing.

The experimental results shown in Figure 14 show that the performance of our proposed multimodal fusion method is better than other methods for each quantitative task. The above results show that our proposed multi-modal fusion method can capture the correlation of high-level features.

### Discriminative Quantitative Analysis

Different from traditional manual feature extraction methods, the features extracted in this paper are automatically learned in image quantization through cascaded neural networks. The gray information on the original image is transformed from shallow to high-level by non-linear transformation of the cascaded neural network, gradually transformed into high-level information with discriminative ability, and finally these expressive features are mapped to disease prediction. In the neural network, the direct display of these “high-level” hidden layer features is usually poor in interpretability. In the quantification of medical images, the neuroimaging is calculated to qualitatively and quantitatively analyze the relevant brain regions affected by AD disease. It is very important to analyze the pathological causes of Alzheimer’s disease and drug research.

Aiming at the problem of poor feature interpretation of deep convolutional neural networks, we try to visualize the areas that have a large impact on quantitative prediction. In order to achieve this goal, we systematically slide the 3D gray cubes on the original image to cover the information of different brain regions, and use the trained model to monitor the changes in the probability output of the network quantification under different masking positions of the original image. If the excluded patch covers the area related to AD, the predicted probability of the correct class will decrease significantly. Therefore, by predicting the change in probability, we can roughly determine the key focus areas that have an impact on AD.

In the experiment, we used MRI and PET images of 10 Alzheimer’s disease patient samples in the test set for visual analysis. First, we select three image blocks with the best overall quantization performance in the two modalities for analysis. Then, each image block uses a 15 × 15 × 15 gray cube block (the gray value is the mean value of the input image block) to slide and mask each area on the image block. Probability prediction is made in the corresponding 3D neural network model. Finally, we calculate the decrease in AD prediction probability after masking to generate the probability change of each sample in each region.

**FIGURE 15:**
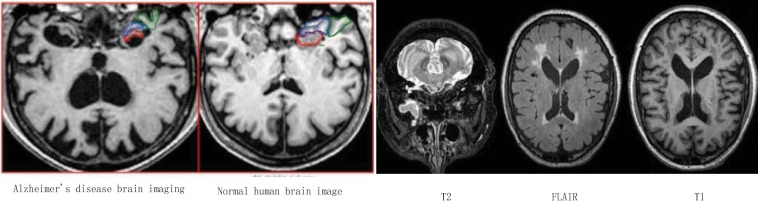
Alzheimer’s disease area that the neural network focuses on.

**FIGURE 16:**
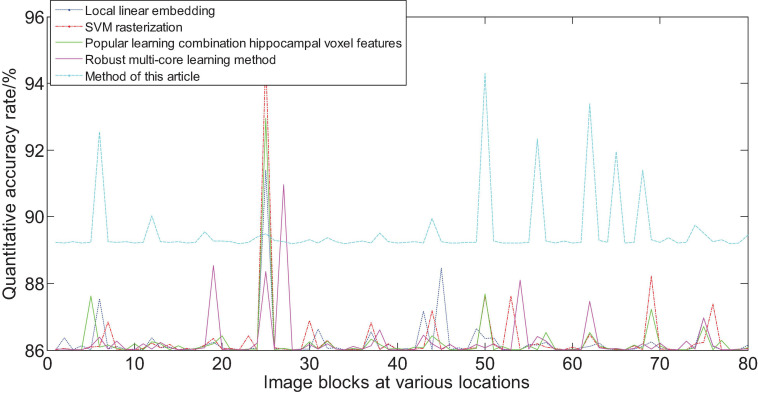
Comparison of the quantification accuracy of the multi-modal cascaded 3D-CNNs proposed in this article and other methods.

Figure 15 shows the area that the neural network focuses on the MRI image. The red part represents the area with the largest probability change, that is, the structural position most relevant to AD. Furthermore, we compare the experimental results of this method with the results of other methods. Figure 16 shows the overall accuracy of the quantification results, comparing the overall results of multimodal quantification for AD vs. NC, p MCI vs. NC, and s MCI vs. NC. It can be seen from the results in the figure that the method proposed in this paper is better than other methods in the quantification of AD vs. NC, and has better sensitivity and greater ROC curve line in MCI vs. NC. The area helps to detect MCI earlier and prevent missed diagnosis. These results once again prove the effectiveness of the method proposed in this paper.

## Conclusion

In this paper, DTI, f MRI, and T1 data are preprocessed and the brain function connection network and the brain white matter structure connection network are respectively constructed. Then the graph theory parameters are introduced into the analysis of the two kinds of brain networks, and the graph theory parameters with significant differences between AD patients and normal people are found as features by T test. This article introduces the calculation principle of the efficient convolution structure-depth separable convolution unit existing in Mobile Net, analyzes its computational complexity and compares it with the traditional convolution layer, and explains that the depth separable convolution unit is in the convolution efficiency in product operations. On this basis, a channel pruning method based on a depth separable convolution unit is presented. The pruning process and the effect of network compression are analyzed with the help of a flowchart. This article is the use of single, parallel and cascaded convolutional neural networks to extract and quantify the experimental process and results of FDG-PET images and MRI. The first is the introduction to the experiment, which describes the sample selection we used for the experiment and the preprocessing work done before the sample is input to the network. The comparative experiment of the evaluation model is introduced, and the experimental results are given to verify the effectiveness of our algorithm framework. This article analyzes the experimental results, and uses the trained model to reversely analyze the affected area of CNN to infer the discriminative brain areas of Alzheimer’s disease. The results prove that the method of combining 2D-CNNs and BGRU proposed in this paper has excellent distinguishing ability for PET monomodal data, indicating that the combination of CNN and RNN can capture the functional change information of brain images. The model of cascaded three-dimensional convolutional neural network can fully utilize the information of multi-modal data while extracting single-modal structural features. The brain image calculation and analysis model proposed in this paper can accurately and effectively quantify the cognitive function of Alzheimer’s disease according to the characteristics of the modal.

## Data Availability Statement

The dataset analyzed for this study can be found in this link. http://adni.loni.usc.edu/.

## Author Contributions

YH and JW contributed equally to this work. All authors contributed to the article and approved the submitted version.

## Conflict of Interest

The authors declare that the research was conducted in the absence of any commercial or financial relationships that could be construed as a potential conflict of interest.
